# Generalized self-calibrating probe approach for CW tissue oximetry

**DOI:** 10.1117/1.JBO.31.9.095001

**Published:** 2026-09-07

**Authors:** Leila Motamed Jahromi, Lin Yang, Alexander von Lühmann, Dirk Grosenick

**Affiliations:** a Physikalisch-Technische Bundesanstalt (PTB), Berlin, Germany; b NIRx Medizintechnik GmbH, Berlin, Germany

**Keywords:** oximetry, spatially resolved spectroscopy, diffusion model, near-infrared spectroscopy

## Abstract

**Significance:**

Functional near-infrared spectroscopy (fNIRS) and oximetry are important noninvasive methods to investigate physiological processes and states in the human brain and muscle tissue. Self-calibrating probe methods combine two light sources with typically two detectors to improve the stability of continuous-wave (CW) devices, but so far are limited to fully symmetric geometries.

**Aim:**

We developed a generalized self-calibrating probe approach to improve accuracy and stability in CW oximetry for arbitrary configurations of two light sources and detectors.

**Approach:**

We used the diffusion theory of light transport to derive the effective attenuation coefficient from self-calibrating probes with arbitrary asymmetric source-detector configurations in reflection. The theory was validated by measurements on tissue-like reference phantoms and by a vascular occlusion test on the forearm muscle of a healthy subject using a dual-wavelength CW fNIRS imager. Results were compared with conventional analysis by spatially resolved spectroscopy (SRS). Time-domain measurements served as a reference.

**Results:**

The phantom studies with the generalized self-calibrating probe approach provided accurate results for effective attenuation and oxygen saturation for the wide variety of source-detector configurations examined. Results from the SRS method showed a slightly larger spread. When submillimeter shifts of the source positions were neglected in the data analysis, large errors were observed in oxygen saturation for both methods. The vascular occlusion test showed good agreement between the self-calibrating approach and the time-domain reference data. Oxygenation profiles from SRS differed strongly across the source-detector combinations, with significant deviations already for the baseline saturation. When CW measurements were analyzed using literature values for the tissue scattering properties, systematic shifts of the oxygen saturation occured. The shifts remain small when the ratio of the reduced scattering coefficients at the two wavelengths of the CW device is close to the true tissue value.

**Conclusions:**

The proposed generalized self-calibrating approach enables quantification of oxygen saturation *in vivo* for a wide variety of source-detector configurations whereby a good estimate of the ratio of the tissue scattering properties at the two wavelengths is required. Our single-case study shows the potential of the self-calibrating approach to considerably improve the accuracy and stability of CW oximetry for the large dynamic range of a vascular occlusion compared with conventional measurements using SRS with a single light source.

## 1 Introduction

Continuous-wave near-infrared spectroscopy (NIRS) is a noninvasive, portable, and versatile imaging technology to investigate dynamic changes of oxy- and deoxyhemoglobin (Hb) concentrations *in vivo*. Due to its simplicity and flexibility, the technique has gained significant attention and wide applications in research and clinical fields. Important applications are functional NIRS of the brain (fNIRS)^1^^–^^4^ and of muscle tissue (mNIRS).^5^^–^^8^ To determine tissue oxygen saturation, which is an important parameter to assess tissue oxygen supply, the absolute concentrations of oxy- and deoxyhemoglobin are needed. Such measurements typically require expensive time-domain or frequency-domain technologies.^9^^–^^11^ To determine oxygen saturation using CW techniques as well, the method of spatially resolved spectroscopy (SRS) has been introduced.^12^ With the minimum configuration of one light source and two detectors with different distances to the source, the effective attenuation of the tissue can be measured at appropriate near-infrared (NIR) wavelengths. To obtain oxygen saturation, reasonable assumptions on the tissue reduced scattering coefficient are required. One option is to use the required reduced scattering coefficients from published time-domain or frequency-domain studies for the respective type of tissue.^5^^,^^13^^–^^15^ In the original SRS method, a linear decrease of scattering versus wavelengths was taken from published time-domain measurements on the brain, and the oxygen saturation calculated under this assumption was referred to as the tissue oxygenation index (TOI).^12^ Another option is the use of multiple wavelengths to gain scattering parameters and scaled chromophore concentrations simultaneously from CW measurements.^16^

For several commercial cerebral oximeters, calibration and validation were done by invasive *in vivo* studies.^17^^–^^19^ Usually, the internal algorithms of commercial oximeters for brain, muscle, or somatic applications are not disclosed. Comparative studies on patients, healthy subjects, and phantoms with different oximeters have revealed significant differences in the oxygenation results.^20^^–^^23^ Near-infrared oximeters are therefore often used to monitor trends rather than to measure absolute oxygen saturation.^24^^,^^25^

The accuracy of SRS measurements is highly sensitive to perturbations of photon propagation caused by probe–tissue coupling variations, local pressure differences, motion artifacts, hair, and superficial pigments. Furthermore, drifts of source intensity or detector sensitivity affect the measured detector signals. All these factors change the detected light intensities independently of tissue physiology. They hamper the correct estimation of the tissue’s effective attenuation and reduce the accuracy of long-term measurements.

Wu et al. applied a self-calibrating method to eliminate the influence of the above factors in a wearable brain oximeter.^13^ The method uses two light sources and two detectors in a strictly symmetric arrangement, with the light sources located at mirror-image distances $\rho_{\text{short}}$ and $\rho_{\text{long}}$ from the two detectors. The SRS measurements for each source can be combined so that the unknown source-to-tissue and tissue-to-detector coupling factors, as well as source intensity and detector sensitivity, are eliminated in the calculation of the effective attenuation. Originally, this type of self-calibrating method was developed for frequency-domain instrumentation to avoid calibration by phantoms.^26^ In practical implementations, the requirement for perfect symmetry limits the flexibility in positioning sources and detectors on the tissue area of interest and, particularly with flexible probes, could be difficult to meet. Probe deformation, positioning errors, and different source positions for the applied wavelengths can introduce small but systematic asymmetries. Wu et al. observed a strong effect of small asymmetries on measured oxygen saturation and developed special procedures to recognize and avoid such asymmetries.^13^ Addressing these inevitable asymmetries is therefore essential to enable accurate, geometry-robust CW oximetry with self-calibrating probe designs. Furthermore, enhanced flexibility in the positioning of light sources and detectors is generally advantageous.

We have developed and validated a novel self-calibrating probe approach for obtaining effective tissue attenuation and oxygen saturation using asymmetric source-detector configurations. Generalized equations allow us to account for the source-detector geometry for any arrangement of two light sources and two detectors. The new method is validated in experiments on tissue-mimicking phantoms employing a CW fNIRS device. In a special series of CW experiments, we investigate very small deviations from perfect symmetry that occur, for example, when using bi-color LEDs. Finally, we demonstrate the application of the new method *in vivo*. A vascular occlusion test on the human forearm muscle is used to compare self-calibrating measurements with the established SRS method for a large range of oxygen saturations. We also investigate the influence of assumptions on the tissue scattering properties for the estimation of oxygen saturation. All CW measurements are validated by state-of-the-art time-domain measurements.

## 2 Theory

The simplest model to describe light propagation in reflectance at the brain is the diffusion theory for the semi-infinite homogeneous medium. For CW light, the outgoing flux density at a distance ρ from the source position is given by^27^

$$J(\rho )=\frac{Sz_{0}}{2\pi }\frac{\;\exp(-\mu_{\mathrm{eff}}\sqrt{\rho^{2}+z_{0}^{2}})}{\left(\rho^{2}+z_{0}^{2}\right)}(\mu_{\mathrm{eff}}+\frac{1}{\sqrt{\rho^{2}+z_{0}^{2}}}).$$ (1)

Here, $\mu_{\mathrm{eff}}=\sqrt{3\mu_{a}\mu_{s}^{\prime }}$ is the effective attenuation coefficient of the medium and $\mu_{s}^{\prime }$ and $\mu_{a}$ are the reduced scattering coefficient and the absorption coefficient. S denotes the number of photons per second injected into the medium. The solution is valid for the zero-boundary condition with the light source assumed at a depth $z_{0}=1/\mu_{s}^{\prime }$ below the surface. Having the detector placed at a distance of a few centimeters from the source, we can use the approximations $(\rho^{2}+z_{0}^{2})\approx \rho^{2}$ and $\mu_{\mathrm{eff}}\gg 1/\sqrt{\rho^{2}+z_{0}^{2}}$ to further simplify Eq. (1):

$$J(\rho )=\frac{Sz_{0}}{2\pi }\frac{\exp(-\mu_{\mathrm{eff}}\rho )}{\rho^{2}}\mu_{\mathrm{eff}}.$$ (2)

Now, we consider the ratio of the signals J for two source-detector distances $\rho_{\text{short}}$ and $\rho_{\text{long}}$:

$$\frac{J(\rho_{\text{short}})}{J(\rho_{\text{long}})}=\exp(-\mu_{\mathrm{eff}}(\rho_{\text{short}}-\rho_{\text{long}}))\frac{\rho_{\text{long}}^{2}}{\rho_{\text{short}}^{2}}.$$ (3)

This equation gives the well-known analytical expression for the effective attenuation:

$$\mu_{\mathrm{eff}}=[\ln(\frac{J\left(\rho_{\text{long}}\right)}{J\left(\rho_{\text{short}}\right)})+2\;\ln(\frac{\rho_{\text{long}}}{\rho_{\text{short}}})]/(\rho_{\text{short}}-\rho_{\text{long}}).$$ (4)

The relation between the measured detector voltages $V_{\text{short}}$ and $V_{\text{long}}$ and the theoretical photon flux densities can be described as $V_{\text{short}}=D_{\text{short}}J(\rho_{\text{short}})$ and $V_{\text{long}}=D_{\text{long}}J(\rho_{\text{long}})$. Here, the factors $D_{\text{short}}$ and $D_{\text{long}}$ consider the sensitivity of the two detectors, including the effective detector area on the surface of the medium, the acceptance angle, and the coupling conditions for the collection of the light. The source parameters such as light power and photon losses due to Fresnel reflection are already contained in the factor S in Eq. (1). When substituting the two flux densities in Eq. (4) with the measured voltages, the ratio $D_{\text{long}}/D_{\text{short}}$ must be known to derive $\mu_{\mathrm{eff}}$.

**Fig. 1:**
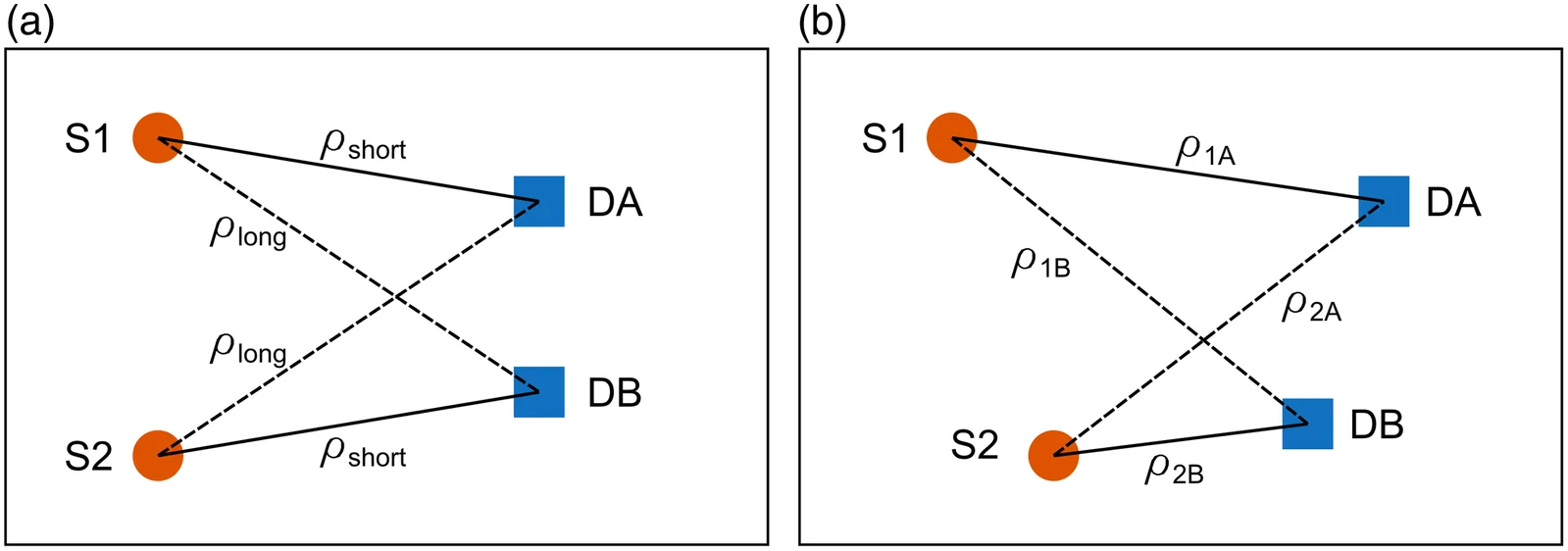
Self-calibrating probe geometry. (a) Symmetric configuration example; (b) asymmetric configuration example. Red circles indicate the sources S1 and S2, blue squares the detectors DA and DB.

In the original self-calibrating probe approach, a second light source is used to form a symmetric source-detector configuration [see example in Fig. 1(a)]. If we denote the sources by subscripts 1 and 2 and the detectors by subscripts A and B, we have four flux densities $J_{1A}$, $J_{1B}$, $J_{2A}$, and $J_{2B}$. Because the number of injected photons (source power and coupling to the medium) can be different for both sources, we replace S in Eqs. (1) and (2) by the source-specific factors $S_{1}$ and $S_{2}$.

Similar to Eq. (3) we consider the ratio $J_{1A}J_{2B}/(J_{1B}J_{2A})$ now, where the short-distance signals are in the numerator, and the long-distance signals are in the denominator. We can substitute the photon fluxes with the measured detector voltages according to $V_{1A}=D_{A}J_{1A}$, $V_{1B}=D_{B}J_{1B}$, $V_{2A}=D_{A}J_{2A}$ and $V_{2B}=D_{B}J_{2B}$ with $D_{A}$ and $D_{B}$ representing the sensitivity factors of both detectors. When we substitute the four fluxes with the four voltages, the detector sensitivity factors cancel out. The source factors $S_{1}$ and $S_{2}$ have also been dropped. This cancellation is the main idea of the self-calibrating probe approach. Using Eq. (2) for each flux, we get:

$$\frac{J_{1A}J_{2B}}{J_{1B}J_{2A}}=\frac{V_{1A}V_{2B}}{V_{1B}V_{2A}}=\frac{\rho_{\text{long}}^{4}}{\rho_{\text{short}}^{4}}\;\exp(-2\mu_{\mathrm{eff}}\left(\rho_{\text{short}}-\rho_{\text{long}}\right)).$$ (5)

The effective attenuation can be derived from this as follows:

$$\mu_{\mathrm{eff}}=[\frac{1}{2}\;\ln(\frac{V_{1B}V_{2A}}{V_{1A}V_{2B}})+2\;\ln(\frac{\rho_{\text{long}}}{\rho_{\text{short}}})]/(\rho_{\text{short}}-\rho_{\text{long}}).$$ (6)

This is the self-calibrating probe result for a symmetric source detector configuration with the two distances $\rho_{\text{short}}$ and $\rho_{\text{long}}$.^26^ Because it does not depend on the source or detector sensitivity factors, it is very robust against hair or skin pigments below an optical element.

For asymmetric configurations [Fig. 1(b)], the distances $\rho_{\text{short}}$ and $\rho_{\text{long}}$ become different for both sources. If we denote the four source-detector distances as $\rho_{1A}$, $\rho_{1B}$, $\rho_{2A}$, and $\rho_{2B}$ now, we get the voltage ratio:

$$\frac{V_{1A}V_{2B}}{V_{1B}V_{2A}}=\frac{D_{A}\frac{S_{1}z_{0}}{2\pi }\frac{\exp(-\mu_{\mathrm{eff}}\rho_{1A})}{\rho_{1A}^{2}}\mu_{\mathrm{eff}}D_{B}\frac{S_{2}z_{0}}{2\pi }\frac{\exp(-\mu_{\mathrm{eff}}\rho_{2B})}{\rho_{2B}^{2}}\mu_{\mathrm{eff}}}{D_{B}\frac{S_{1}z_{0}}{2\pi }\frac{\exp(-\mu_{\mathrm{eff}}\rho_{1B})}{\rho_{1B}^{2}}\mu_{\mathrm{eff}}D_{A}\frac{S_{2}z_{0}}{2\pi }\frac{\exp(-\mu_{\mathrm{eff}}\rho_{2A})}{\rho_{2A}^{2}}\mu_{\mathrm{eff}}}.$$ (7)

Again, all sensitivity factors cancel out, and we can write:

$$\frac{V_{1A}V_{2B}}{V_{1B}V_{2A}}=\exp(-\mu_{\mathrm{eff}}(\rho_{1A}-\rho_{1B}+\rho_{2B}-\rho_{2A}))\frac{\rho_{1B}^{2}\rho_{2A}^{2}}{\rho_{1A}^{2}\rho_{2B}^{2}}.$$ (8)

This equation can be rearranged to obtain $\mu_{\mathrm{eff}}$:

$$\mu_{\mathrm{eff}}=[\ln(\frac{V_{1B}V_{2A}}{V_{1A}V_{2B}})+2\;\ln(\frac{\rho_{1B}\rho_{2A}}{\rho_{1A}\rho_{2B}})]/(\rho_{1A}-\rho_{1B}+\rho_{2B}-\rho_{2A}).$$ (9)

Equation (9) represents the self-calibrating probe expression for asymmetric source-detector configurations.^28^ It can be applied to all geometries for which the denominator in Eq. (9) is nonzero. This condition is equivalent to the requirement that the (sign-sensitive) path differences $\rho_{1A}-\rho_{1B}$ and $\rho_{2A}-\rho_{2B}$ between the two detectors must be different for the two sources:

$$\rho_{1A}-\rho_{1B}\neq \rho_{2A}-\rho_{2B}.$$ (10)

If the denominator in Eq. (9) becomes zero, then the voltage ratio in Eq. (8) does not depend on $\mu_{\mathrm{eff}}$.

In practice, the different voltages $V_{\mathrm{SD}}$ in Eq. (9) exhibit a certain noise level $\Delta V_{\mathrm{SD}}$, and the source-detector distances $\rho_{\mathrm{SD}}$ have an uncertainty $\Delta \rho_{\mathrm{SD}}$. From Eq. (9), we can estimate the relative uncertainty of $\mu_{\mathrm{eff}}$ using error propagation:

$$\frac{\Delta \mu_{\mathrm{eff}}}{\mu_{\mathrm{eff}}}=\frac{1}{\mu_{\mathrm{eff}}\Delta R}\sqrt{\underset{SD}{\sum }\left[{\left(\frac{\Delta V_{\mathrm{SD}}}{V_{\mathrm{SD}}}\right)}^{2}+{\left(\frac{2}{\rho_{\mathrm{SD}}}+\mu_{\mathrm{eff}}\right)}^{2}\Delta \rho_{\mathrm{SD}}^{2}\right]}.$$ (11)

Here, the sum runs over the four source-detector combinations 1A, 1B, 2A, and 2B. Equation (11) can also be used to estimate the uncertainty of $\mu_{\mathrm{eff}}$ for the conventional SRS method with a single source [Eq. (4)]. Then, $\rho_{\mathrm{SD}}$ and $V_{\mathrm{SD}}$ have to be replaced by $\rho_{\text{short}}$, $\rho_{\text{long}}$ and $J(\rho_{\text{short}})$, $J(\rho_{\text{long}})$. The effective distance ΔR denotes the denominator in Eq. (9) or Eq. (4).

Self-calibrating method:

$$\Delta R=\rho_{1A}-\rho_{1B}+\rho_{2B}-\rho_{2A}.$$ (12a)

SRS method:

$$\Delta R=\rho_{\text{short}}-\rho_{\text{long}}.$$ (12b)

Equation (11) shows the expected behavior that low individual detector signals $V_{\mathrm{SD}}$ being typical for large distances will increase the uncertainty of $\mu_{\mathrm{eff}}$. Furthermore, small values of ΔR enlarge the uncertainty of $\mu_{\mathrm{eff}}$ for both the self-calibrating and the SRS method.

The application of the self-calibrating method for arbitrary source-detector configurations fulfilling the inequality (10) is not limited to the diffusion model used with the zero boundary condition. The advantage of the zero boundary condition is that an analytical expression for $\mu_{\mathrm{eff}}$ can be specified. If we look at any other model for the photon flux

$$J(\rho )=f(\mu_{\mathrm{eff}},\rho ),$$ (13)

the ratio in Eq. (7) can be written more generally as

$$\frac{V_{1A}V_{2B}}{V_{1B}V_{2A}}=\frac{f(\mu_{\mathrm{eff}},\rho_{1A})f(\mu_{\mathrm{eff}},\rho_{2B})}{f(\mu_{\mathrm{eff}},\rho_{1B})f(\mu_{\mathrm{eff}},\rho_{2A})}.$$ (14)

To derive $\mu_{\mathrm{eff}}$ here, Eq. (14) can be solved numerically. In this way, for example, the diffusion model with an extrapolated boundary condition or a Monte Carlo simulation of light propagation can be applied.

## 3 Experimental Methods

### 3.1 Investigations on Phantoms

**Fig. 2:**
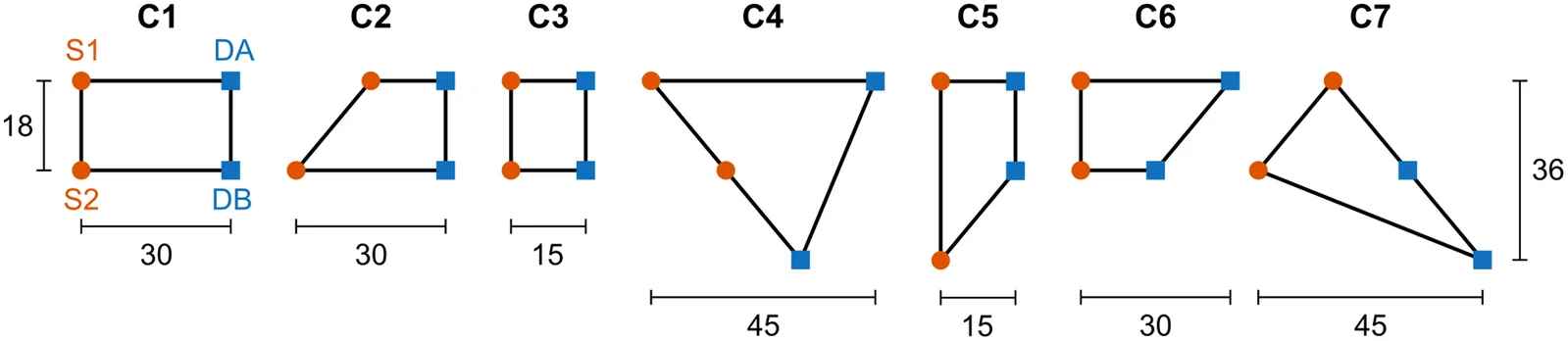
Symmetric (C1, C3) and asymmetric experimental configurations. Red circles indicate the sources, and blue squares represent the detectors. Lengths are given in mm.

The experimental validation of the asymmetric probe approach was done on several phantoms and by a vascular occlusion test using a NIRSport2 CW Brain Imager (NIRx Medizintechnik GmbH, Germany). This device was equipped with 16 bi-color LED sources (760 and 850 nm) and 16 silicon photodiode detectors (SiPD). When applied for fNIRS on the head, these sources and detectors are fixed at designated positions of a flexible cap. For phantom studies, we selected two sources and two detectors to realize the two symmetric and five asymmetric configurations shown in Fig. 2. The sources and detectors were inserted into the corresponding holes of a black plastic plate with a(x,y) grid of 15  mm×18  mm step size. The rectangular configuration C1 is taken as the symmetric basis configuration with source-detector distances of 30 and 35 mm. These distances are large enough for the light to reach either the brain or muscle through the overlying tissue layers. The phantom face of the plastic plate was covered by 2 mm thick black sponge rubber to reduce light traveling along the surface of the phantom. The data acquisition software of the device was modified to store the voltages of the two detectors for source S1 and source S2 for both wavelengths. Signals were recorded at a rate of 7.63 Hz. The LED power was typically between 1 and 2 mW at each wavelength. For each configuration, the source power at both wavelengths was chosen such that the signal for the shortest source-detector distance was about 700 mV (for phantoms) or 500 mV (*in vivo* at baseline) to avoid detector saturation. The detector dark noise level was below 60  μV. The SRS method [Eq. (4)] requires that both detectors have the same sensitivity. Measurements on a phantom with the detectors being placed sequentially at the same position showed relative deviations by a few percent. Therefore, measured voltages were corrected accordingly before calculating the effective attenuation $\mu_{\mathrm{eff}}$ at both wavelengths.

To obtain the absorption coefficients of the phantoms from the CW measurements, we apply the equation

$$\mu_{a}(\lambda )=\mu_{\mathrm{eff}}^{2}(\lambda )/(3\mu_{s}^{\prime }\left(\lambda \right)).$$ (15)

In this step, we use the reference values of the reduced scattering coefficients $\mu_{s}^{\prime }$ of the phantoms, given below. The absorption spectrum of the phantom is defined by the black absorber added during manufacturing. To investigate the potential of the CW method for tissue oximetry, we assume that the phantom absorption is caused by oxy- and deoxyhemoglobin and by water instead, which are the main tissue absorbers at the two NIR wavelengths. With this assumption, we can estimate a virtual oxygen saturation value. To this end, we solve the two equations

$$\mu_{a}(\lambda_{1,2})=(\varepsilon_{\mathrm{HbR}}\left(\lambda_{1,2}\right)c_{\mathrm{HbR}}+\varepsilon_{\mathrm{HbO}2}\left(\lambda_{1,2}\right)c_{\mathrm{HbO}2})\ln10+\kappa_{H2O}\mu_{a,H2O}(\lambda_{1,2}),$$ (16)

with $\lambda_{1}=760\;\mathrm{nm}$ and $\lambda_{2}=850\;\mathrm{nm}$ to obtain the virtual concentrations of oxyhemoglobin ($c_{\mathrm{HbO}2}$) and deoxyhemoglobin ($c_{\mathrm{HbR}}$). $\varepsilon_{\mathrm{HbR}}$, and $\varepsilon_{\mathrm{HbO}2}$ are the tabulated molar extinction coefficients of deoxy- and oxyhemoglobin. To be close to *in vivo* conditions, a volume fraction of water (absorption coefficient $\mu_{a,H2O}$) of $\kappa_{H2O}=75\%$ is included here as a small correction. From the two hemoglobin concentrations, we determine the oxygen saturation by its definition:

$${\mathrm{StO}}_{2}=c_{\mathrm{HbO}2}/(c_{\mathrm{HbR}}+c_{\mathrm{HbO}2}).$$ (17)

**Fig. 3:**
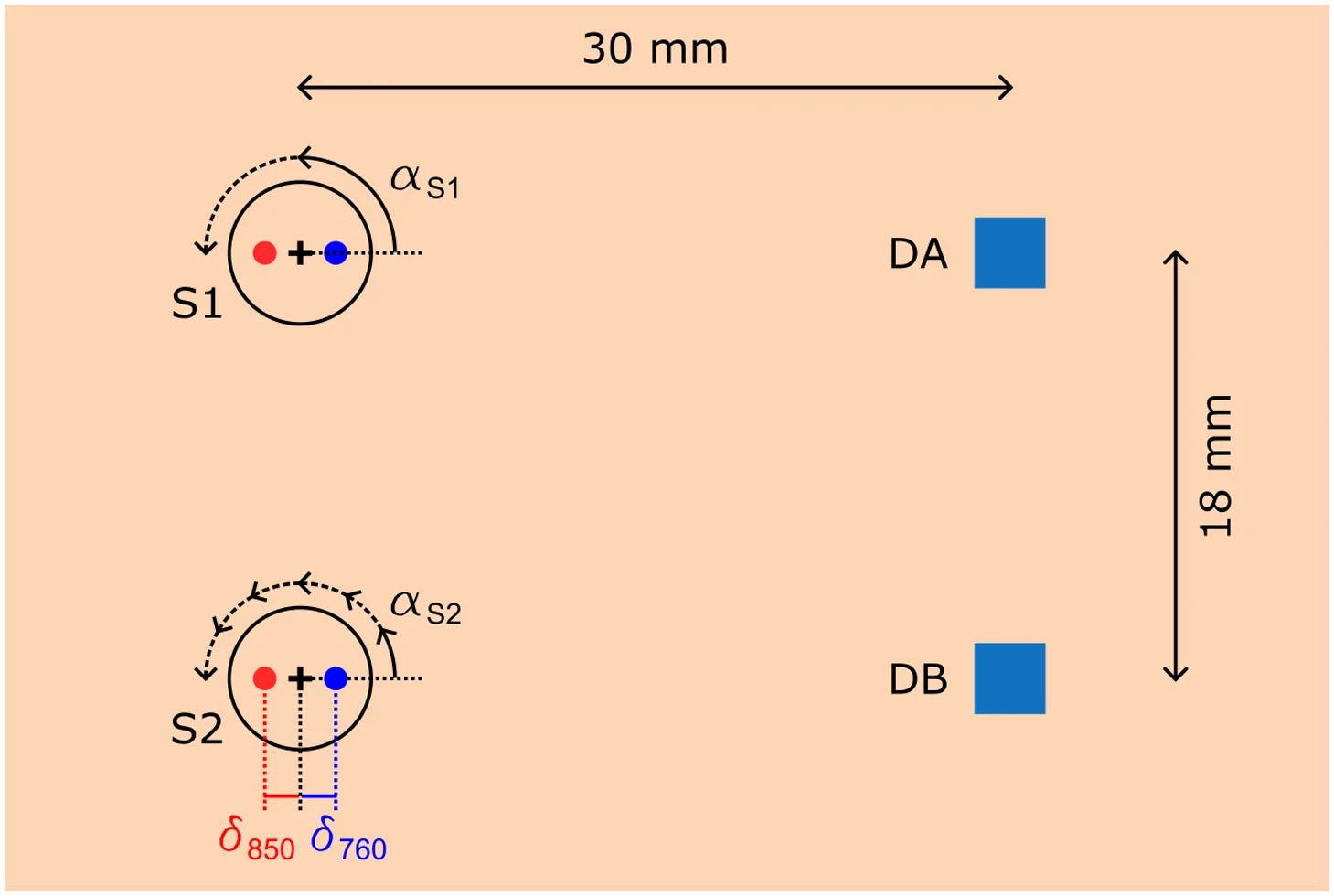
Local rotation of source S1 light spots (90 deg steps) and source S2 spots (30 deg steps) on the surface of the phantom for configuration C1 (blue circles: 760 nm spots, red circles: 850 nm spots, DA and DB indicate detectors A and B).

The symmetric configuration C1 was used for further investigations. The light spots of the bi-color LED at 760 and 850 nm are located at slightly different positions on the surface of the phantom. By rotating the sources S1 and S2 in their holders, a slight asymmetry was introduced because the positions of the two light spots on the surface of the phantom were varied (Fig. 3). With these experiments, the effects of small positional errors on optical properties and oxygen saturation were tested.

### 3.2 Reference Phantoms

The phantoms were made from polydimethylsiloxane (PDMS) with titanium oxide particles as scatterers and black toner as an absorber (BioPixS Limited, Ireland). They have a cylindrical shape (diameter 9.4 cm and height 5 cm). The CW optical probe was attached to the top of the flat side. Prior to the investigations with the NIRSport 2 device, the optical properties of these phantoms were determined from time-domain (TD) measurements between 690 and 900 nm (step size 10 nm) using a femtosecond Ti:Sapphire laser (MaiTai HP, Spectra Physics, California, United States). A microchannel-plate photomultiplier (R3809U-51, Hamamatsu Photonics) and time-correlated single photon counting (SPC150-NX, Becker & Hickl GmbH) were employed to record distributions of times of flight of photons (DTOFs) in reflectance geometry with a source-detector distance of 2 cm. Graded-index optical fibers were used to ensure a high timeresolution. The half-width of the instrumental response functions was about 70 ps. Absorption and reduced scattering coefficients were obtained by interpreting the measurements with a Monte Carlo model of light propagation for the semi-infinite homogeneous medium. Hereby, the instrumental response functions were taken into account. The spectrum of the reduced scattering coefficients was smoothed by modeling it with the power law $\mu_{s}^{\prime }(\lambda )=\mu_{s}^{\prime }(\lambda_{0})(\lambda /\lambda_{0}{)}^{-b}$ and the smoothed coefficients were used in a second fit to get the absorption coefficients. Table 1 summarizes the optical properties that serve as reference values for the CW experiments. The (virtual) oxygen saturation values in the right column of Table 1 were obtained by applying Eqs. (16) and (17).

**Table 1:** Reference optical properties of PDMS phantoms (from time-domain measurements). Uncertainties are ±5% relative for $\mu_{s}^{\prime },\mu_{a},\mu_{\mathrm{eff}}$, and ±1% absolute for ${\mathrm{StO}}_{2}$ (estimated from reproducibility measurements).

Phantom	$\mu_{s}^{\prime }/{\mathrm{cm}}^{-1}$		$\mu_{a}/{\mathrm{cm}}^{-1}$		$\mu_{\mathrm{eff}}/{\mathrm{cm}}^{-1}$		${\mathrm{StO}}_{2}$ (%)^a^
	760 nm	850 nm	760 nm	850 nm	760 nm	850 nm	
B3	9.89	8.51	0.0996	0.101	1.72	1.60	55.7
B5	9.87	8.49	0.197	0.191	2.42	2.20	57.5
B7	10.5	9.08	0.306	0.295	3.10	2.83	59.0

a ${\mathrm{StO}}_{2}$ obtained under the assumption that $\mu_{a}$ at the two wavelengths is caused by oxy- and deoxyhemoglobin (with a hypothetical water volume fraction of 75%).

### 3.3 In Vivo Investigations

**Fig. 4:**
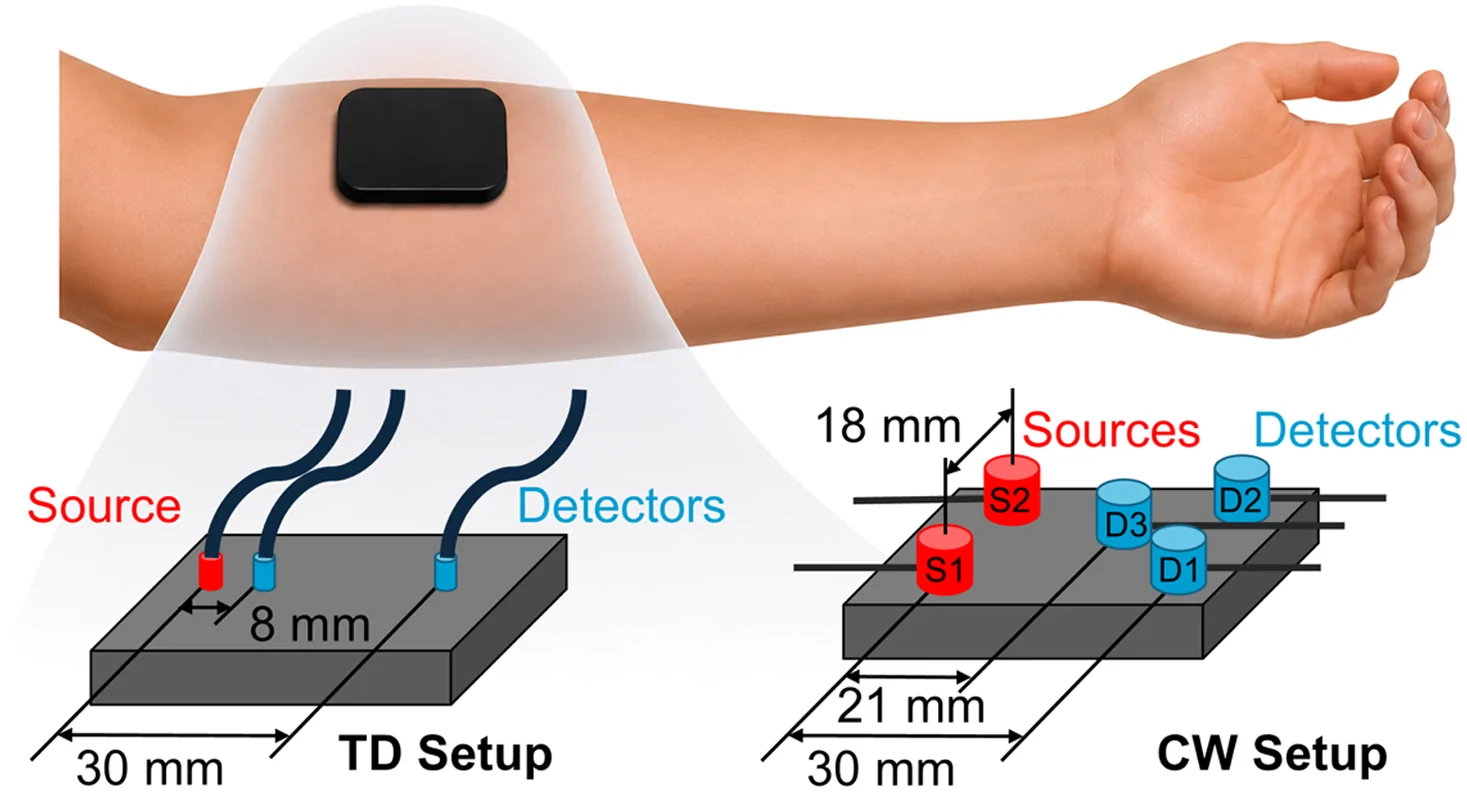
Probe configuration and probe positioning on the forearm for the vascular occlusion test with the CW fNIRS imager (self-calibrating and SRS method) and with the TD reference method.

To demonstrate the benefits of the generalized self-calibrating probe approach *in vivo*, a proof-of-principle investigation was done on the brachioradialis muscle of the left forearm of a healthy male subject (64 years old). The investigation was approved by the PTB Ethics Committee, and written informed consent was obtained from the subject. To vary the oxygen saturation over a larger range, a vascular occlusion test (3 min duration) was used. The occlusion experiment was initially conducted with a time-domain (TD) method serving as a reference. After a recovery time of 3 h the experiment was repeated with the CW NIRSport2 device. For each investigation, a manually inflated cuff was placed on the left upper arm. The probe for either the time-domain or the CW measurement was positioned on top of the brachioradialis muscle (Fig. 4). Each investigation started with a 1 min baseline period. The cuff was then inflated to a pressure of 240 mmHg within a few seconds to induce an arterial occlusion. After a 3 min occlusion period, the cuff was deflated, and data collection continued for another 7 min to record the recovery phase. The probe and cuff were then removed.

The probe for the TD measurement (Fig. 4) holds a source fiber and two detection fibers (source-detector distances 8 and 30 mm). A ps white light laser (SuperK Fianium FIU-15, NKT Photonics, Bregnerødvej 144, 3460 Birkerød, Denmark) was coupled to an acousto-optic filter to switch through eight wavelengths (658, 685, 727, 760, 802, 830, 850, and 900 nm) within 1.4 s. For each wavelength, distributions of times of flight of photons (DTOFs) were recorded at the two source-detector distances with an exposure time of 100 ms. Two different hybrid photomultipliers (HPM-100-07C at 8 mm distance, HPM-100-50 at 30 mm distance, Becker & Hickl GmbH) were connected to time-correlated single photon counting electronics (SPC150-NX and SPC150, Becker & Hickl GmbH, Nunsdorfer Ring 7-9, 12277 Berlin, Germany). Graded-index optical fibers were used to obtain a high time-resolution (half-width of the instrumental response function between 41 ps (658 nm) and 31 ps (900 nm) for detector HPM-100-07C and between 166 ps (658 nm) and 123 ps (900 nm) for detector HPM-100-50).

DTOFs measured during the 1 min baseline period were summed up and analyzed by the semi-infinite medium Monte Carlo model described above for the phantom measurements. For analysis of the individual DTOFs, the scattering coefficient at each wavelength was fixed to the baseline value, since changes in scattering during the occlusion were considered negligible. Oxygen saturation was calculated from the optical properties at 760 and 850 nm to apply the same wavelengths as available in the CW measurements. The time courses of oxygen saturation at 30 and 8 mm distance were smoothed using a five-point sliding average corresponding to a time interval of 7 s.

The vascular occlusion was repeated with the CW NIRSport2 device, employing two sources and three detectors. The probe configuration is illustrated in Fig. 4. Sources S1 and S2, together with detectors D1 and D2, form the same rectangular configuration as used in the phantom experiments. When combining detector D3 with either D1 or D2, asymmetric configurations are obtained instead. Conventional SRS configurations are given using one of the sources with two of the detectors. Detector voltages were recorded at a rate of 7.63 Hz. The analysis of the CW *in vivo* data follows the scheme given above for the phantom measurements. The required reduced scattering coefficients were taken from the time-domain *in vivo* results at 30 mm source-detector distance. Alternative analyses were done with the time-domain result for 8 mm and with values from the literature (see Sec. 4; Table 2). The time courses of oxygen saturation were smoothed using a five-point sliding average corresponding to a time interval of 655 ms.

**Table 2:** Oxygen saturation values for TD reference and for the various CW configurations in Fig. 12.

Method	Detector	Oxygen saturation / %						
		Baseline^a^	Min^b^	Δ(Min) ^c^	Max^b^	Δ(Max) ^c^	Recovery^d^	Δ(Recover) ^e^
TD reference	30 mm	66.9	54.0	12.9	73.1	6.2	67.3	0.4
Self-calibration sym.	D1D2	69.1	54.6	14.5	77.5	8.4	70.6	1.5
Self-calibration asym.	D2D3	69.1	59.0	10.1	76.2	7.1	71.1	2
Self-calibration asym.	D1D3	69.0	49.4	19.6	78.8	9.8	69.8	0.8
SRS source S1	D1D2	59.0	36.4	22.6	73.4	14.4	60.8	1.8
SRS source S1	D2D3	63.8	43.3	20.5	73.0	9.2	64.9	1.1
SRS source S1	D1D3	67.7	50.4	17.3	73.2	5.5	68.7	1
SRS source S2	D1D2	75.6	65.4	10.2	80.7	5.1	77.4	1.8
SRS source S2	D2D3	57.9	17.9	40.0	69.8	11.9	57.7	−0.2
SRS source S2	D1D3	68.1	51.1	17.0	75.7	7.6	69.0	0.9

a Average over period 0 … 60s;

b Minimum and maximum values of the graphs in Fig. 12;

c Δ(Min), Δ(Max) give the differences between columns Min, Max, and column Baseline;

d Values at t=600  s in Fig. 12 (recovery);

e Difference between columns Recovery (t=600  s) and Baseline.

## 4 Results and Discussion

### 4.1 Asymmetric Configurations

**Fig. 5:**
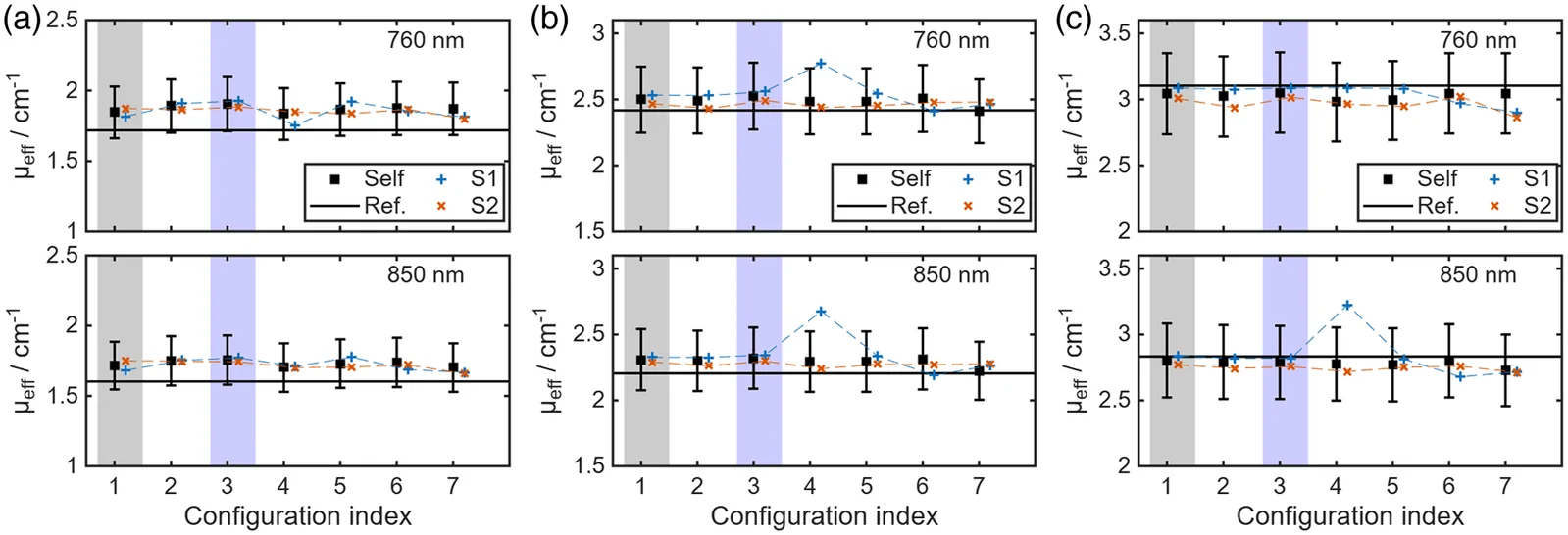
Effective attenuation $\mu_{\mathrm{eff}}$ obtained by the self-calibration method [black squares—Eq. (9)] and by using either source S1 or S2 for SRS analysis [crosses—Eq. (4)]. Configuration index refers to the seven probe configurations in Fig. 2; (a) phantom B3 ($\mu_{a}\approx 0.1\;{\mathrm{cm}}^{-1}$), (b) phantom B5 ($\mu_{a}\approx 0.2\;{\mathrm{cm}}^{-1}$), (c) phantom B7 ($\mu_{a}\approx 0.3\;{\mathrm{cm}}^{-1}$). The horizontal lines indicate the reference properties from Table 1. The two symmetric configurations, C1 and C3, are highlighted by the colored background.

Measured voltages for the seven source-detector configurations in Fig. 2 were analyzed by the self-calibrating method [Eq. (9)]. For comparison with the SRS method, data were also analyzed by Eq. (4). The latter analysis was conducted for both sources. Figure 5 illustrates the resulting effective attenuation for the three PDMS phantoms with reference absorption values of about 0.1, 0.2, and $0.3\;{\mathrm{cm}}^{-1}$, covering the typical absorption range for brain and muscle tissue. The coefficients obtained by the self-calibrating method are almost identical for the asymmetric and symmetric geometries. Variations are smaller than 2%, showing that Eq. (9) accounts very well for asymmetries. The effective distance ΔR [Eq. (12)] varies between about 5 mm (configuration C7) and 24 mm (configuration C5). Configuration C7 demonstrates that an effective distance of 5 mm is sufficiently large even for very large individual source-detector distances ($\rho_{1B}$ and $\rho_{2B}$ are about 47 and 48 mm here). The relative uncertainty of $\mu_{\mathrm{eff}}$ from Eq. (11) varies for this configuration between 5% (phantom B3) and 10% (phantom B7). For the other configurations, it is typically 2%.

The attenuation coefficients from the SRS method are close to those of the self-calibrating approach. Differences between both sources are smaller than 4% except for configuration C4, where several strong deviations occur for source S1. For this configuration, the effective distance ΔR is only 1.9 mm, which is obviously too small here for reliable measurements on the phantoms B5 and B7, which have higher absorption. The relative uncertainty of $\mu_{\mathrm{eff}}$ from Eq. (11) becomes about 10% in these cases compared with uncertainties around 2% to 3% for the other SRS configurations. A detailed analysis of the various contributions in Eq. (11) shows that the small value of ΔR is the main reason for the large uncertainty. In comparison, effective distances of 5 mm (being present here in configurations C1 and C2) yield stable results, similar to the self-calibrating method. Thus, ΔR should not fall significantly below 5 mm when source-detector distances in the 30 to 45 mm range are used.

Figure 5 shows systematic deviations between the CW and the TD method. To better assess these deviations, the small error bars given by Eq. (11) were scaled up to 10%. For the phantom with the lowest absorption, B3 [Fig. 5(a)], $\mu_{\mathrm{eff}}$ is overestimated by about 7% compared with the reference values. In the case of the higher-absorbing phantom B5 [Fig. 5(b)], the overestimation is reduced to about 3%, whereas for the highest absorption [Fig. 5(c)], an underestimation of $\mu_{\mathrm{eff}}$ by about 2% is observed. These systematic deviations are independent of the chosen symmetric or asymmetric probe configuration, indicating a more general deviation between the CW method and the time-domain reference method.

**Fig. 6:**
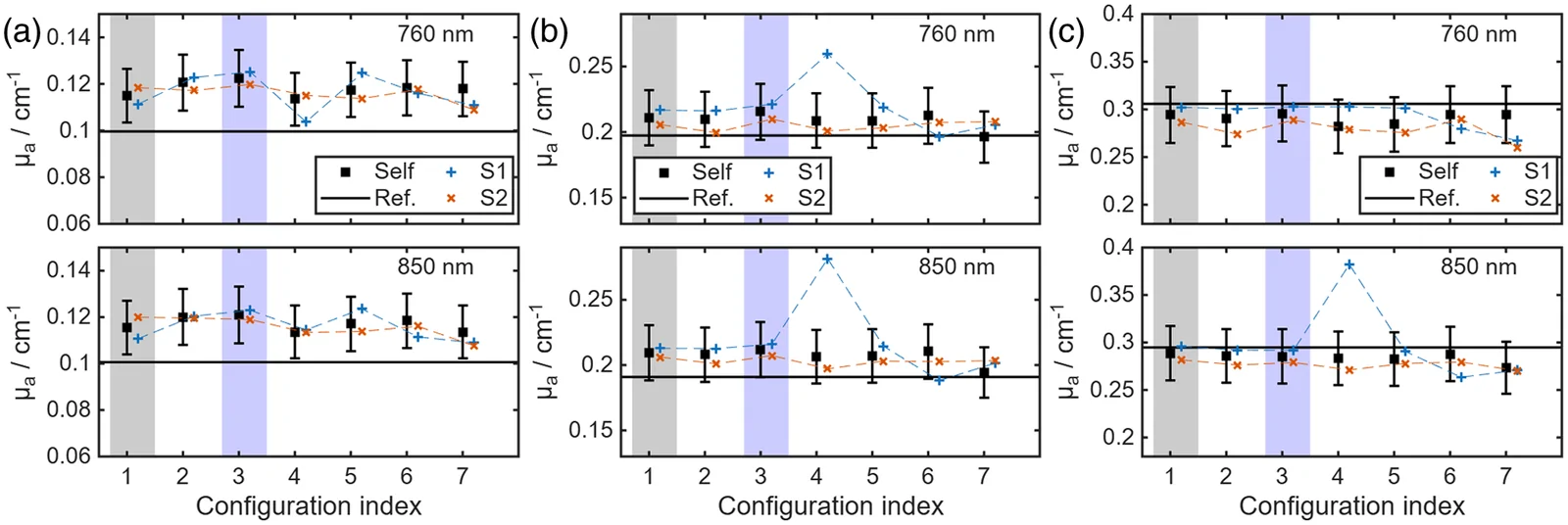
Absorption coefficients $\mu_{a}$ were derived from the effective attenuation coefficients in Fig. 5 using the reference values of the reduced scattering coefficients as prior knowledge. Data refer again to the seven configurations in Fig. 2; (a) phantom B3 ($\mu_{a}\approx 0.1\;{\mathrm{cm}}^{-1}$), (b) phantom B5 ($\mu_{a}\approx 0.2\;{\mathrm{cm}}^{-1}$), (c) phantom B7 ($\mu_{a}\approx 0.3\;{\mathrm{cm}}^{-1}$). The horizontal lines indicate the reference absorption coefficients from Table 1. Results are shown for the self-calibrating method and for the SRS method using either source S1 or S2.

The estimation of the oxygen saturation from the effective attenuation at the two wavelengths [Eqs. (15) to (17)] requires knowledge of or assumptions on the related reduced scattering coefficients. To be as accurate as possible here, we use the known reference values of $\mu_{s}^{\prime }$ from the time-domain method to further evaluate the asymmetric configurations. The influence of assumptions on $\mu_{s}^{\prime }$ will be discussed below for the *in vivo* investigation. Figure 6 shows the resulting absorption coefficients for the seven source-detector configurations. Like $\mu_{\mathrm{eff}}$, the absorption from the self-calibrating method is practically the same in asymmetric and symmetric configurations. The relative deviation of $\mu_{a}$ from the reference values is larger because the absorption scales with the square of $\mu_{\mathrm{eff}}$ [Eq. (15)]. In the case of phantom B3 [Fig. 6(a)], the 10% error bars no longer cover the reference absorption. For the other two phantoms [Figs. 6(b) and 6(c)], the absorption is still within the 10% error bars. The turn from overestimation to underestimation with increasing nominal absorption remains the same as for the effective attenuation in Fig. 5. The results for the SRS method show a larger relative deviation between the two sources than for effective attenuation, which in turn is due to the fact that absorption scales with the square of $\mu_{\mathrm{eff}}$.

**Fig. 7:**
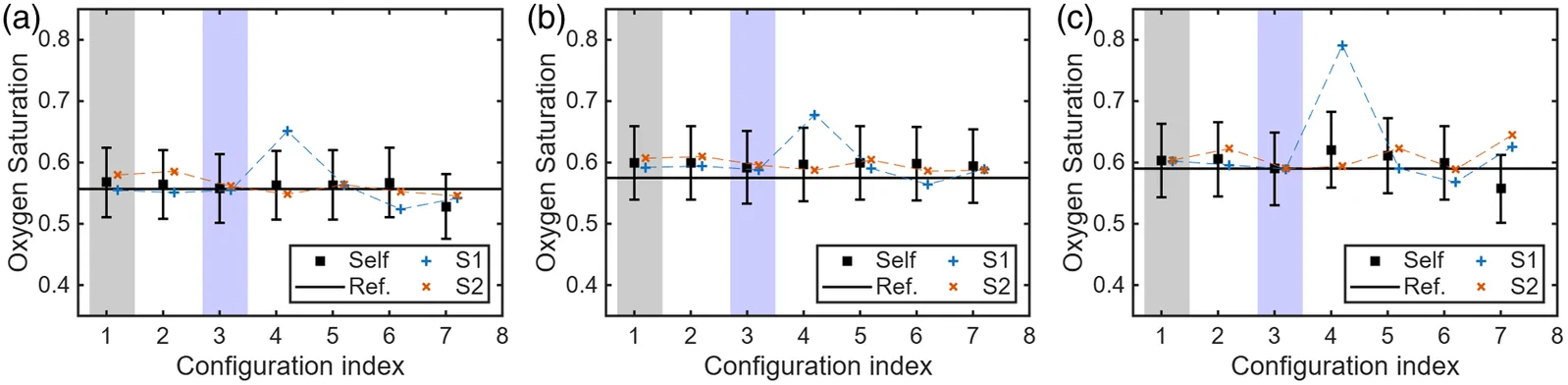
Oxygen saturation for the self-calibrating and the SRS method calculated from the absorption coefficients in Fig. 6; (a) phantom B3 ($\mu_{a}\approx 0.1\;{\mathrm{cm}}^{-1}$), (b) phantom B5 ($\mu_{a}\approx 0.2\;{\mathrm{cm}}^{-1}$), (c) phantom B7 ($\mu_{a}\approx 0.3\;{\mathrm{cm}}^{-1}$). The horizontal lines indicate the reference oxygen saturation values from Table 1.

Figure 7 illustrates the oxygen saturation values for the three phantoms. For the self-calibrating method, the results are stable over the different probe configurations. Furthermore, for all three phantoms, the oxygen saturation is close to the reference values. The (absolute) deviation is about 1% in Fig. 7(a) and up to 3% in Figs. 7(b) and 7(c). The error bars indicate a 10% relative deviation. For configuration 7, a slightly larger deviation from the reference values occurs in Figs. 7(a) and 7(c). This configuration contains the largest source-detector distances (up to 48.5 mm), which lead to very low detector voltages and increased noise. The saturation values from the SRS method differ for both sources by up to 4% absolute saturation. In the case of configuration C4, estimation of saturation fails for source S1, in accordance with the large error for $\mu_{\mathrm{eff}}$ and $\mu_{a}$.

**Fig. 8:**
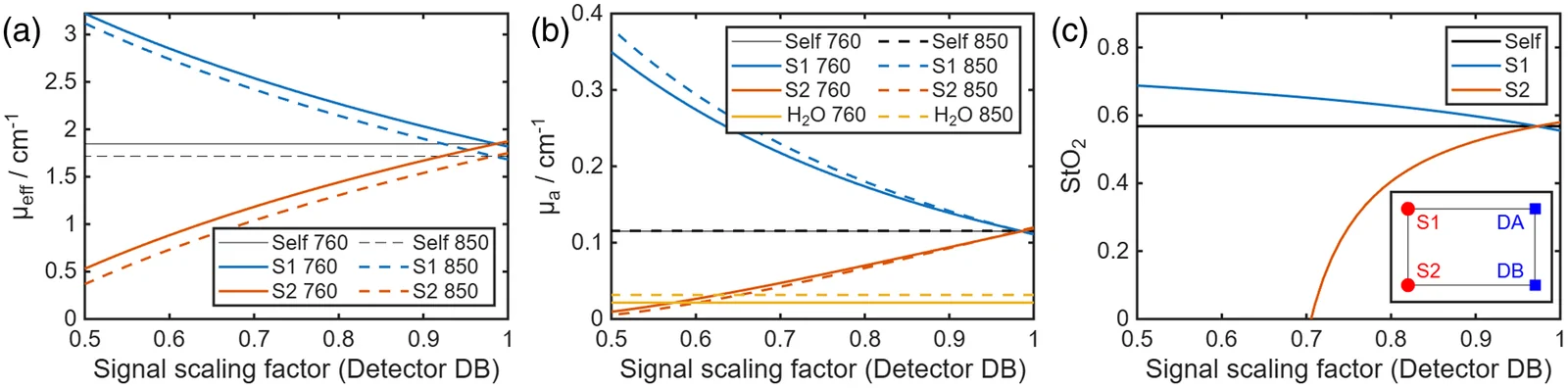
(a) Change of effective attenuation, absorption, and oxygen saturation for the self-calibrating method (Self) and for SRS (sources S1 and S2) when the signal of detector DB is reduced to up to 50% of the original value at both wavelengths (760 and 850 nm). Water absorption in (b) is given for $\kappa_{H2O}=0.75$. The inset in (c) shows the source-detector configuration (cf. C1 in Fig. 2).

The generally better agreement of oxygen saturation with the reference values compared with $\mu_{a}$ and $\mu_{\mathrm{eff}}$ is due to the fact that the saturation is determined by the ratio of the absorption values at the two wavelengths. Systematic deviations in absorption are thus partially compensated in the ratio formation. Overall, the phantom experiments confirm that the generalized self-calibrating method correctly describes symmetric and asymmetric source-detector configurations. The conventional SRS method yields similar results as long as the source-detector distances are below 4 cm. This result is expected because the phantom experiment has been done under perfect conditions. During *in vivo* investigations, detector signals could be disturbed dynamically by small movements or statically by local skin pigments or by hair, reducing the signal of the affected detector. To assess the effect of such perturbations, Fig. 8 illustrates changes in $\mu_{\mathrm{eff}}$, $\mu_{a}$ and oxygen saturation when the signal of one detector is reduced. Data refers to the measurement on phantom B3 in rectangular configuration C1 with the signal attenuation applied to detector DB being closest to source S2 (see inset in Fig. 8). Results for the other configurations are similar.

As given by Eq. (9) the displayed quantities are not affected when the self-calibrating method is used. Because the detector with the reduced signal is the short-distance detector for source S2, $\mu_{\mathrm{eff}}$ and $\mu_{a}$ for this source decrease with decreasing detector signal when the SRS method is applied. A signal reduction to 80% results already in a decrease of $\mu_{a}$ by about 40%. When the signal is further decreased, absorption approaches the assumed water absorption ($\kappa_{H2O}=0.75$). The effect on oxygen saturation is dramatic; the saturation quickly goes down to zero. The change in oxygen saturation for source S1 is moderate because the large overestimation of absorption by SRS is similar for both wavelengths and the influence of water absorption decreases. This simulation indicates the strong benefit of the self-calibrating method for *in vivo* investigations compared with conventional measurements by SRS.

### 4.2 Source Rotation Effects

**Fig. 9:**
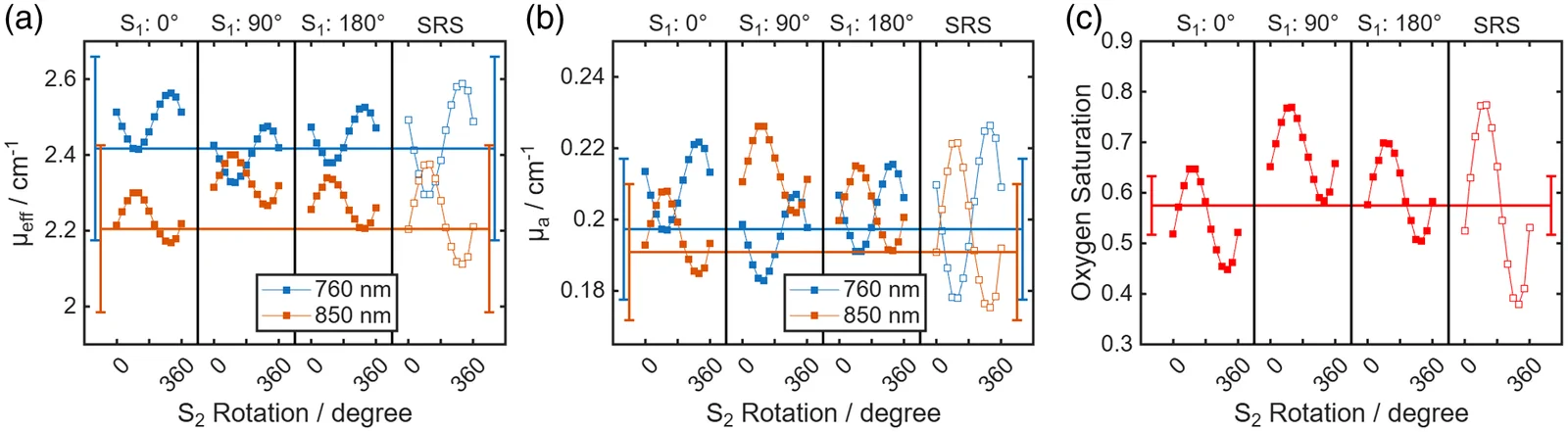
(a) Effective attenuation $\mu_{\mathrm{eff}}$, (b) absorption coefficients $\mu_{a}$, and (c) virtual oxygen saturation ${\mathrm{StO}}_{2}$ for phantom B5 obtained for rotation of source S2 when the small deviation between the 760 and 850 nm emission points is neglected. Self-calibrating method: solid squares (source S1 at three orientations: 0 deg, 90 deg, and 180 deg); SRS method: open squares. The horizontal lines indicate the reference values from Table 1 (with error bars of 10%, representing the measurement uncertainty of Table 1 expanded by a factor of 2).

The applied bi-color LED chips emit the two wavelengths through a common lens. This design means that the two light beams have slightly different directions and hit the phantom surface at different locations. To study the significance of the small distance between the two emission points at the phantom surface, we rotated source 2 in the symmetric configuration C1 with steps of 30 deg. The experiments were done for three different orientations of source S1 (see Fig. 3). Figure 9 shows the results for effective attenuation, the absorption coefficients, and the oxygen saturation under the assumption that the two light beams coincide at the same spot. With this assumption, Eq. (6) can be applied for analysis by the self-calibrating method. Data in Fig. 9 refer to phantom B5.

All three quantities show a sinusoidal dependency on the rotation angle of source 2. For the self-calibrating method, the orientation of source S1 shifts the patterns systematically along the vertical axis. The effective attenuation varies moderately within the plotted ±10% error bar of the reference values. The amplitude of the variation increases for the absorption coefficient because it goes with the square of $\mu_{\mathrm{eff}}$ [Eq. (15)], but it is not far outside the 10% deviation interval of the reference. However, the resulting oxygen saturation deviates by up to 25% (relative) from the reference value. Depending on the source orientation, one gets saturation values between 0.44 and 0.78 compared with the reference value of 0.58. The vertical spread of the sinusoidal dependencies in Fig. 9 becomes even larger when the SRS method is applied.

The strong variation of oxygen saturation for both analysis methods is caused by the approximately 180 deg phase shift in the underlying effective attenuation for the two wavelengths. Maximum attenuation at 760 nm coincides with minimum attenuation at 850 nm and vice versa. The reason for the phase shift is obvious: the 760 and 850 nm emission points at the phantom surface are on opposite sides of the rotation center.

**Fig. 10:**
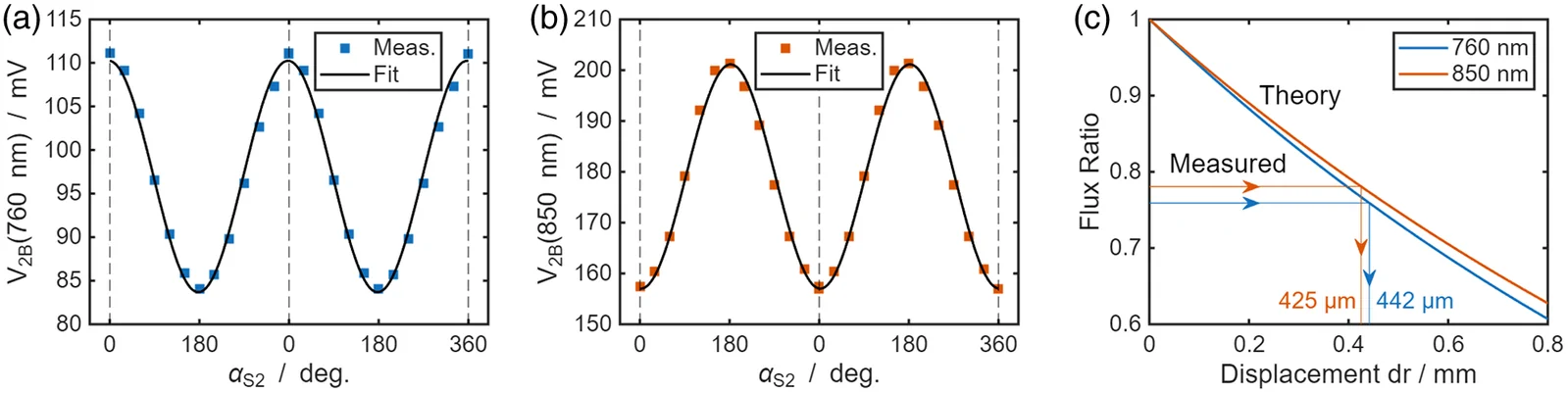
Detector voltages for phantom B5 at (a) 760 nm and (b) 850 nm for rotation of source S2 (solid squares). Maximum signals are obtained at 2.1 deg (760 nm) and 177.9 deg (850 nm) from fitting with a cosine function; (c) Theoretical flux ratio J(r+dr)/J(r-dr) for phantom B5 as a function of displacement dr from the center of rotation (r=3  cm). Arrow lines indicate the estimation of the displacement from the measured voltage ratio $V_{\min}/V_{\max}$ at both wavelengths.

To determine the position of the light spots on the phantom surface with respect to the center of the source (the rotation center), we fitted the measured voltages at both wavelengths as a function of the source rotation angle with a cosine function [Figs. 10(a) and 10(b)]. The angular positions of the maximum voltages indicate the rotation angles at which the emission points at the two wavelengths are closest to detector DB (cf. Fig. 3). Moreover, to obtain the displacement of the emission points from the center of the source at the surface of the phantom, we determined the experimental ratio of the minimum and maximum voltages, $V_{\min}/V_{\max}$. This ratio was compared with theoretical ratios J(r+dr)/J(r-dr) with J representing the photon flux density from diffusion theory [Eq. (2)] for the semi-infinite medium [Fig. 10(c)]. Here, dr represents the small deviation of the emission point from the rotation center, and r is the distance of the source rotation point to the detector (3 cm in the example of Fig. 10). For source S2, shown in Fig. 10(c), we obtain displacements dr=443  μm at 760 nm and 426  μm at 850 nm. Source S1 is a little bit more asymmetric with dr=410  μm (760 nm) and 485  μm (850 nm).

**Fig. 11:**
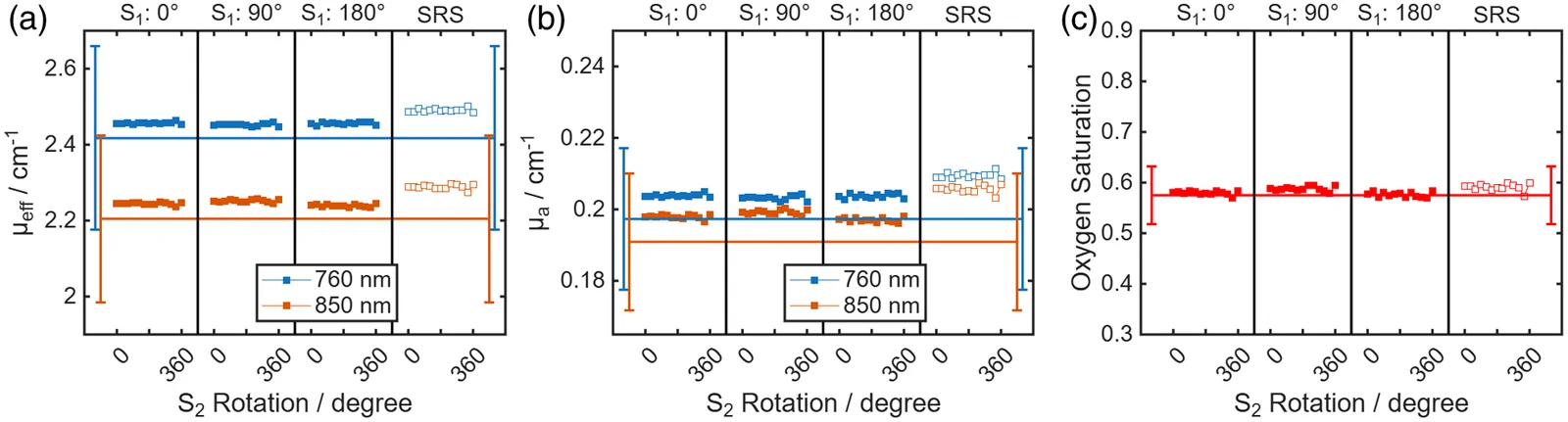
(a) Effective attenuation $\mu_{\mathrm{eff}}$, (b) absorption coefficients $\mu_{a}$, and (c) virtual oxygen saturation ${\mathrm{StO}}_{2}$ for phantom B5 obtained for rotation of source S2 when the true positions of the emission points are taken into account by Eqs. (9) or (4). Self-calibrating method: solid squares (source S1 at three orientations: 0 deg, 90 deg, and 180 deg); SRS method: open squares. The horizontal lines indicate the reference values from Table 1 (with error bars of 10%, representing the measurement uncertainty of Table 1 expanded by a factor of 2).

Figure 11 shows the results for effective attenuation, absorption, and oxygen saturation as a function of the rotation angle of source S2 when the determined displacements dr of the emission points on the phantom surface are considered in Eq. (9) (self-calibration method) and Eq. (4) (SRS method). Attenuation and absorption coefficients do not depend on the rotation angle now. Oxygen saturation for the self-calibrating method corresponds perfectly to the reference value with a relative deviation of up to 2%. Deviation from the reference for the SRS method is only slightly larger. Effective attenuation and absorption coefficients show a slight overestimation with respect to the reference values of about 2% and 4%, respectively, in accordance with Figs. 5(b) and 6(b). For the SRS method, the deviation is about twice as large. Because the data at both wavelengths deviate in the same direction, the errors are compensated when oxygen saturation is derived.

The oxygen saturation results underscore the need to account for the different positions of the two LED spots (760 and 850 nm) on the surface of the phantom, even though they are only about 800  μm apart. The strong oscillations of $\mu_{\mathrm{eff}}$ and $\mu_{a}$ in Fig. 9 are caused mainly by the rectangular geometry (configuration C1). When source S2 is rotated, the difference $\rho_{2A}-\rho_{2B}$ in the denominator of Eq. (9) for the self-calibrating method and $\rho_{\text{short}}-\rho_{\text{long}}$ in Eq. (4) for the SRS method oscillate between about 4.7 and 5.1 mm for both wavelengths. This oscillation will be strongly reduced when the two detectors and the source are arranged in a straight line. Then both distances are changed almost in phase during source rotation, and their difference is nearly constant. In the self-calibrating method, a fully linear arrangement results in an extended probe length, and the sampled volumes for the two sources would hardly overlap compared with the rectangular design.

Overall, the presented generalized model enables us to transfer the application of the self-calibrating probe method in tissue oximetry to arbitrary geometries and helps to enhance the accuracy of the resulting oxygen saturation values. The phantom experiments have shown improved stability and accuracy compared with SRS measurements with a single light source.

### 4.3 Vascular Occlusion

**Fig. 12:**
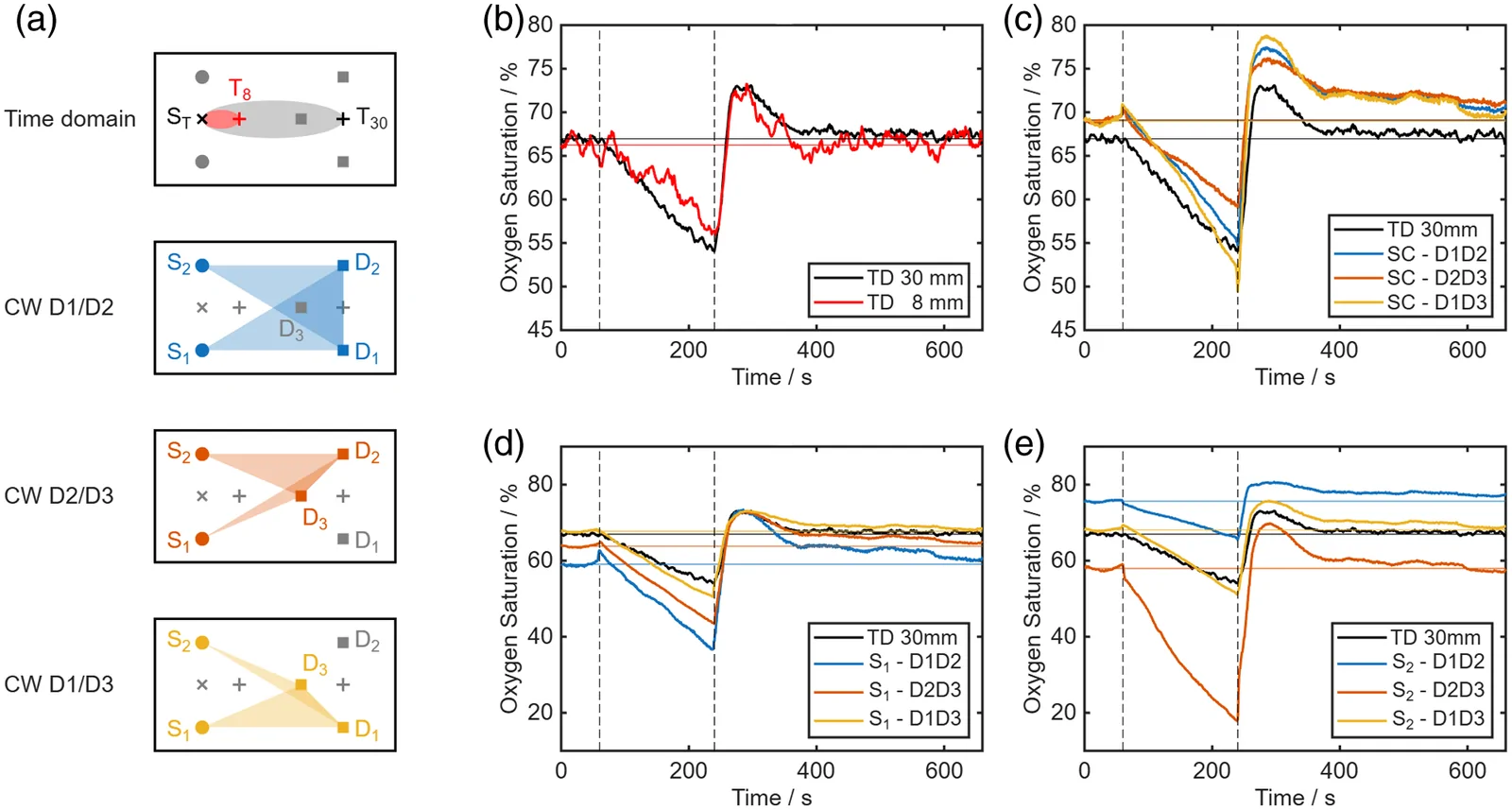
(a) Source-detector configurations for vascular occlusion measurements; (b) oxygen saturation from time-domain measurements with 30 and 8 mm source-detector distance; (c) oxygen saturation from self-calibrating (SC) measurements (D1D2 = symmetric, D2D3 and D1D3 = asymmetric) compared with time-domain at 30 mm; (d) oxygen saturation from SRS measurements with source S1; (e) oxygen saturation from SRS measurements with source S2. Line colors in (b) to (e) refer to detector colors in probe schemes (a).

In this section, we apply the generalized self-calibration method for the analysis of oxygen saturation in the forearm muscle during the vascular occlusion and compare it with the conventional SRS method. Figure 12(a) illustrates the probe geometry for the TD reference and the CW measurements. For each CW configuration, the longer source-detector distance amounts to 35 mm; the shorter one is either 30 mm or 21 mm. Thus, the penetration depth should be comparable to the TD measurement at a 30 mm distance.

Figure 12(b) shows the oxygen saturation time course obtained from the TD measurements at 30 mm, which serves as the reference for the CW results. During the 3 min occlusion, saturation drops from 66.9% to 54.0%. Table 2 summarizes the main parameters of the temporal profiles in Fig. 12 (baseline saturation, minimum value, maximum value, saturation after recovery (data point at t=600  s in Fig. 12), and changes with respect to the original baseline). The oxygen saturation profile for the TD measurement with an 8 mm distance, which samples superficial tissue regions, is very similar. Thus, the decrease in saturation is about the same at different depths within the tissue region measured by the TD probe. The oxygen saturation profile at 8 mm is noisier due to the short photon pathlengths because the effect of absorption changes on the shape of the measured DTOFs is smaller compared with the 30 mm distance.

Figure 12(c) summarizes the results for the CW self-calibrating measurements. The three configurations yield the same baseline value, which is 2% higher than the TD reference. The change in saturation at the end of the occlusion varies between 10% and 20% (absolute change in saturation), whereby the symmetric configuration (14.5%) is closest to the TD reference (12.9%). The saturation overshoot after the occlusion is larger (7.1% to 9.8%) than in the TD measurement (6.2% with respect to the baseline). The saturation after recovery from the occlusion is about 1% to 2% larger than the baseline saturation at the beginning. Because TD and CW methods sample slightly different tissue volumes, results could differ due to tissue heterogeneity. Furthermore, data for both methods were recorded in different occlusion measurements. In addition, interpretation with the homogeneous model could result in deviations, e.g., due to differences in the depth sensitivity between TD and CW methods.

The results for the conventional SRS analysis of the CW data are much more heterogeneous [Figs. 11(d) and 11(e)]. Each detector pair yields a different saturation profile. Only the measurements with detector pair D1/D3 follow the TD reference. This is the case for both sources, whereby different parts of the tissue volume are covered. For source S1 [Fig. 12(d)], the spread for the three SRS measurements is still moderate. When source S2 is used, saturation values for detector pair D1/D2 are generally too high. Even the baseline value appears very large. In contrast, for detector pair D2/D3 the baseline saturation becomes too small (only 58%). And the drop by 40% during the occlusion looks unrealistically high. In the case of the symmetric detector pair D1/D2 the self-calibrating method yields the average of both single source profiles. For unsymmetric geometries, there is no averaging effect by the self-calibrating method. Overall, the results for the SRS method appear heterogeneous. Because the different source-detector combinations sample partially different tissue volumes, the variations could reflect spatial tissue heterogeneities. Furthermore, the SRR method is very sensitive to variations in the coupling of the sources and detectors to the tissue. The device characteristics should not contribute to the large spread of results, because phantom measurements have shown that the sensitivity of all detectors is the same. Overall, for the probe configurations examined in this single-subject study, the self-calibrating method shows better agreement with the TD reference data than the conventional SRS method.

Up to here, oxygen saturation for CW measurements was always calculated by using the reduced scattering coefficients from the TD reference measurements. Thus, errors due to wrong tissue scattering properties were avoided. For stand-alone CW measurements, approximate scattering properties must be taken from published time-domain or frequency-domain data. In the following, we reanalyze the CW *in vivo* measurements using scattering properties from Matcher et al. for forearm tissue,^29^ and by Damagatla et al. for the lower forearm as well as for muscle tissue of the upper arm.^30^ We also apply the scattering coefficients from our 8 mm TD measurement, representing the overlying tissue (skin and fat on top of the forearm muscle). Table 3 lists the values of the various reduced scattering coefficients.

**Table 3:** Reduced scattering coefficients for analysis of the CW *in vivo* measurements.

Analysis		$\mu_{s}^{\prime }/{\mathrm{cm}}^{-1}$		Source of $\mu_{s}^{\prime }$
No.	Code Fig. 13	760 nm	850 nm	
1	CW30	8.07	7.71	Our time-domain measurement (30 mm distance)
2	Matcher	7.12	6.67	Matcher et al.^29^ $\mu_{s}^{\prime }(\lambda )=(11-5.1\times 10^{-3}\times \lambda /\mathrm{nm})\;{\mathrm{cm}}^{-1}$
3	CW08	8.87	7.99	Our time-domain measurement (8 mm distance)
4	Dam. FA	6.32	5.56	Damagatla et al.^30^ (forearm radius ulna position)
5	Dam. UA	8.37	7.75	Damagatla et al.^30^ (upper arm muscle position)

**Fig. 13:**
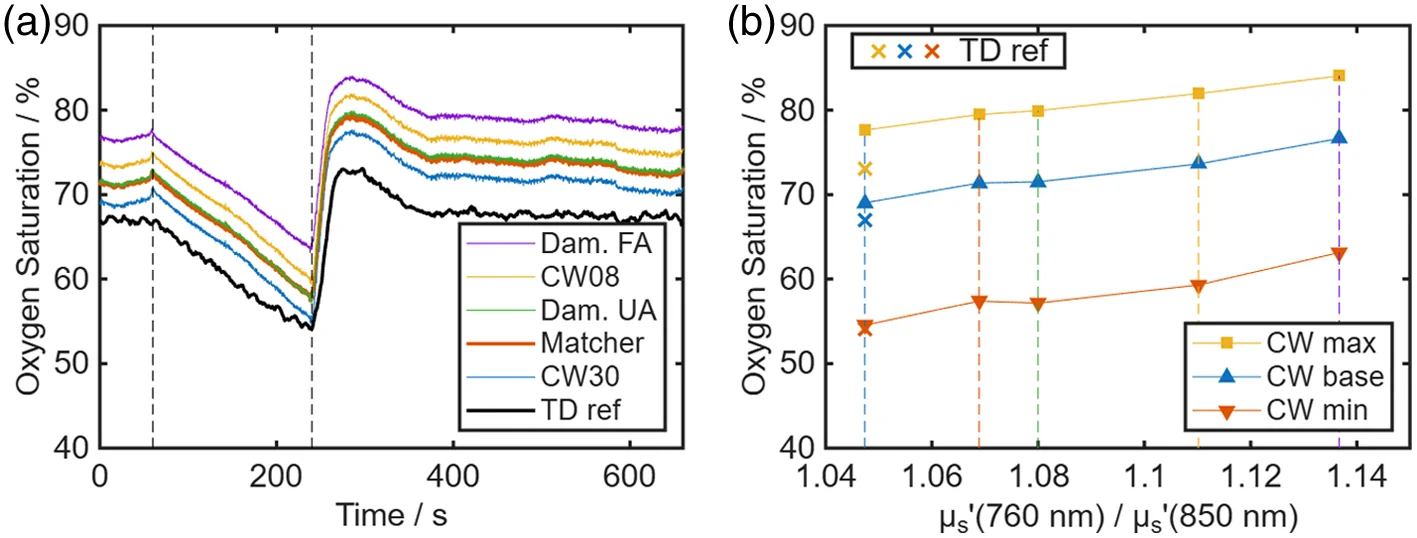
(a) Oxygen saturation temporal profiles (vascular occlusion) obtained for the 5 sets of reduced scattering coefficients listed in Table 3 (legend refers to the abbreviations in Table 3, *TD ref* is the TD reference profile at 30 mm, profiles *Matcher* and *Dam*. UA are almost on top of each other); (b) change of oxygen saturation versus ratio of $\mu_{s}^{\prime }$ at both wavelengths for baseline (CW base), at the minimum (CW min), and at the maximum (CW max) of the vascular occlusion temporal profile in (a). The color of the dashed vertical lines corresponds to the legend in (a).

Figure 13(a) shows the temporal profiles for the symmetric self-calibrating geometry [detectors D1/D2 in Fig. 12(a)]. Analysis sets 2 to 5 of Table 3 shift the profiles to higher saturation values, whereby the drop by the occlusion remains stable at about 14% as obtained with the true tissue scattering properties in Fig. 12(c) (denoted as profile CW30 here). The smallest shifts (about a 2% increase compared with profile CW30) are observed with the forearm data from Matcher et al. and the upper arm muscle data by Damagatla et al. When the skin-dominated properties from our TD measurements are applied, the profile shifts up by two more percent. In addition, the strongest deviation occurs with the bone-dominated forearm dataset of Damagatla et al.

Figure 13(b) illustrates that the saturation (baseline, minimum, and maximum values) increases approximately linearly with the ratio of the reduced scattering coefficients at the two wavelengths. If this ratio is close to the true value for the subject under investigation, then the saturation should be most accurate. Overall, when using literature values of reduced scattering coefficients in the CW self-calibrating probe approach, which are representative of muscle tissue, oxygen saturation temporal profiles are obtained that do not deviate more than 4% from the time-domain reference in our vascular occlusion test.

## 5 Conclusion

We have developed a novel self-calibrating method suitable for a wide variety of configurations consisting of two light sources and two detectors to measure the effective attenuation with CW light and to determine tissue oxygen saturation. A prerequisite for the applicability of this method is that the magnitude of the effective distance in Eq. (12) should not fall significantly below 5 mm. The method was successfully validated by investigations on tissue-mimicking phantoms and by a vascular occlusion test on muscle tissue of the forearm using a CW fNIRS brain imager that allows for free positioning of sources and detectors. For both types of investigation, reference values were obtained by accompanying TD measurements.

The vascular occlusion test demonstrated a considerably higher stability of the self-calibrating method compared with conventional SRS measurements. Oxygen saturation of the muscle tissue should be essentially independent of the specific position of the measurement, because metabolic activity during the vascular occlusion test is expected to be constant. The self-calibrating method confirmed this expected behavior. Baseline values of oxygen saturation were independent of the selected detector pair. Both symmetric and asymmetric geometries yielded the same saturation. Moreover, the temporal profiles of oxygen saturation were similar, with moderate differences in the minimum saturation at the end of the occlusion period and at the overshoot maximum after the start of reperfusion. Compared with the TD reference measurement, self-calibrating temporal profiles were shifted to larger saturation values by about 2% absolute saturation. Results on saturation from the conventional SRS method are very heterogeneous. They depend on the source position and on the chosen detector pair. Baseline saturation varies between 59% and 76% compared with the 67% TD reference value and the 69% baseline saturation for self-calibration. Moreover, the decline during the occlusion varies from 10% to 40% absolute change of saturation. Thus, the SRS method provided a more qualitative picture of the dynamics of oxygen saturation here, while the self-calibration approach yielded dynamic behavior similar to that of the TD reference measurement. Our results are based on a single-case study. Further investigations are needed to assess interindividual variability, reproducibility, and the effects of tissue heterogeneity.

CW oximetry needs assumptions on the scattering properties of the tissue. When replacing the true reduced scattering coefficients from the reference method in this study with literature values for forearm/muscle tissue, a shift of the temporal profiles to higher saturations by about 2% was obtained. Extended sets of scattering properties showed that the shift is determined by the ratio of the scattering coefficients at the two wavelengths used here.

Differences between the self-calibration and the SRS method in the phantom investigations were visible but relatively small. This is the expected behavior because probe-to-tissue coupling is stable and the phantom is homogeneous compared with the heterogeneous structure of the forearm tissue. A simulation on a reduced detector signal to account for potential perturbations under *in vivo* conditions revealed severe problems of the single-source SRS method, guiding to strongly underestimated absorption and oxygen saturation values up to a breakdown of the saturation result. In contrast, the self-calibrating method was unaffected. The phantom experiments also showed some systematic deviations of the effective attenuation derived from the CW measurements with respect to the TD reference method. A set of source rotation experiments with bi-color LEDs demonstrated that neglecting submillimeter-scale shifts in light source positions can lead to large errors in oxygen saturation if these shifts have opposite signs for the two wavelengths. This behavior occurs for the self-calibrating method as well as for the conventional SRS method when rectangular or other area-like arrangements are applied.

In conclusion, our *in vivo* proof-of-principle investigation has shown that, compared with the established SRS method, the generalized self-calibrating approach has the potential to significantly improve the accuracy and stability of CW oximetry across the wide dynamic range associated with a vascular occlusion. The advantages of self-calibration are important for *in vivo* investigations in which conventional SRS measurements are sensitive to motion artifacts, to changes in light coupling, to shadowing of the detector by local hair or skin pigments, or to superficial vascular structures. The generalized method enables high flexibility in source-detector positioning. With the CW self-calibration method, quantitative measurements of oxygen saturation should be feasible in the various application scenarios on brain or muscle tissue when a good estimate for the ratio of the reduced scattering coefficients at the two wavelengths is applied.

## Data Availability

The data that support the findings of this study are available from the corresponding author upon reasonable request.
